# *Stenotrophomonas pavanii* MY01 induces phosphate precipitation of Cu(II) and Zn(II) by degrading glyphosate: performance, pathway and possible genes involved

**DOI:** 10.3389/fmicb.2024.1479902

**Published:** 2024-10-23

**Authors:** Shengchen Zhao, Zitong Xu, Jihong Wang

**Affiliations:** ^1^College of Resource and Environmental Science, Jilin Agricultural University, Changchun, Jilin, China; ^2^Key Laboratory of Straw Biology and Utilization, Ministry of Education, Jilin Agricultural University, Changchun, Jilin, China

**Keywords:** glyphosate, heavy metal, gene, molecular docking, ANN model

## Abstract

Microbial bioremediation is an advanced technique for removing herbicides and heavy metals from agricultural soil. In this study, the strain *Stenotrophomonas pavanii* MY01 was used for its ability to degrade glyphosate, a phosphorus-containing organic compound, producing PO_4_^3−^ as a byproduct. PO_4_^3−^ is known to form stable precipitates with heavy metals, indicating that strain MY01 could potentially remove heavy metals by degrading glyphosate. Therefore, the present experiment induced phosphate precipitation from Cu(II) (Hereinafter referred to as Cu^2+^) and Zn(II) (Hereinafter referred to as Zn^2+^) by degrading glyphosate with strain MY01. Meanwhile, the whole genome of strain MY01 was mined for its glyphosate degradation mechanism and its heavy metal removal mechanism. The results of the study showed that the strain degraded glyphosate best at 34°C, pH = 7.7, and an inoculum of 0.7%, reaching 72.98% within 3d. The highest removal of Cu^2+^ and Zn^2+^ in the test was 75.95 and 68.54%, respectively. A comparison of strain MY01’s genome with glyphosate degradation genes showed that protein sequences GE000474 and GE002603 had strong similarity to glyphosate oxidoreductase and C-P lyase. This suggests that these sequences may be key to the strain’s ability to degrade glyphosate. The GE001435 sequence appears to be related to the phosphate pathway, which could enable phosphate excretion into the environment, where it forms stable coordination complexes with heavy metals.

## 1 Introduction

The rapid expansion of agriculture has raised a major environmental issue: heavy metal pollution of farmland (Zhao et al., 2019). Removing heavy metals from the environment is extremely difficult and challenging (Lai et al., 2023). Heavy metals can accumulate in living organisms, posing a risk to human health through the food chain (Medyńska-Juraszek et al., 2022; Yadav et al., 2019). Heavy metals are common pollutants in the environment, such as Pb, Cd, Cr, Cu, Zn, etc. (Chang et al., 2023). While copper (Cu) and zinc (Zn) are essential micronutrients, concentrations above certain thresholds (around 100 mg Cu/kg and 600 mg Zn/kg in neutral soil) can harm the environment (Tóth et al., 2016). Copper-contaminated soils have become very common in areas of organic agricultural production, due to the use of Cu-based fungicides, such as Bordeaux mixture, to control plant diseases (Dao et al., 2021). The increasing use of ZnO particles in glass, ceramics, lubricants, paints, and plastics, etc., has been associated with the inhibition of the growth of *E. coli* bacteria, and they can be hazardous to human health (Khan et al., 2021). Therefore, from the perspective of human health and environmental protection, remediation of Cu and Zn contaminated soil is of great significance.

Using microorganisms to remove heavy metals is an eco-friendly approach that poses minimal risk of secondary contamination. Microorganisms can eliminate heavy metals through various mechanisms, such as chelation, adsorption, reduction, and mineralization (Lin et al., 2023; Pan et al., 2023; Yi et al., 2022). Microbial-induced heavy metal precipitation has received increasing attention over the past decade due to its high efficiency and environmental friendliness (Huang et al., 2019; Pang et al., 2021). Microbial mineralization and immobilization of heavy metals primarily occur through microbial metabolism, which generates phosphate (PO_4_^3−^) or carbonate (CO_3_^2−^). Phosphate and carbonate are common ligands that form stable coordination compounds with heavy metals. They achieve this through coordination bonds, as both have multiple negatively charged oxygen atoms that can provide lone pairs of electrons to bind with the metal, resulting in stable compounds (Zeng et al., 2017). This method produces heavy metal minerals that are large in particle size, immobile, and structurally stable. Consequently, bioremediation technology holds significant potential for use in soil heavy metal remediation.

In addition to heavy metals, herbicides are also pollutants that seriously affect the soil environment of agricultural fields (Cabral et al., 2023). Currently, glyphosate, as a widely used herbicide, has become one of the important agricultural tools (Venditti et al., 2023). Glyphosate is a potent, broad-spectrum herbicide effective against many weeds. However, continuous use can lead to its accumulation in farmland, causing environmental pollution. While glyphosate breaks down quickly in soil, it can still impact the soil’s microbial community (Adrian et al., 2024). In addition, the high water solubility of glyphosate enables it to spread along with water flow, it is thus widespread in rivers and groundwater, and its average concentration detected is 2–430 μg/L, which is higher than standard concentration of the largest pollutant in drinking water, at 0.7 μg/L (Chen et al., 2022). Continuous application of glyphosate can lead to its accumulation in farmland, causing environmental pollution. Excessive glyphosate runoff into water bodies may also affect aquatic organisms (An et al., 2023; Tajai et al., 2023). Scribner (Geng et al., 2021) found that glyphosate and its byproducts are more toxic than initially thought. In addition, glyphosate is also lethal to non-target plants and therefore may have negative impacts on wild plants and ecosystems (Zioga et al., 2022). Therefore, it becomes crucial to explore the potential of microorganisms in degrading glyphosate.

A number of strains have been identified that are capable of degrading glyphosate, including *Agrobacterium radiobacter*, *Enterobacter cloacae*, *Pseudomonas pseudomallei*, and others (Chen et al., 2022). There are two main ways in which microorganisms degrade glyphosate (Chen et al., 2022). One of them is the conversion of glyphosate to glyoxylic acid and Aminomethyl phosphonic acid by glyphosate oxidoreductase, then to phosphorylformaldehyde by transminase, and finally to PO_4_^3−^ and formaldehyde by phosphatase. Another process involves the participation of C-P hydrolase in the degradation, leading to the production of PO_4_^3−^. This pathway is similar to the common dephosphorylation pathway. The degradation of glyphosate by any means can produce PO_4_^3−^. If the strain itself possesses the phosphate pathway, it can form stable ligand compounds with heavy metals. This method can simultaneously remove heavy metals and glyphosate from farmland, which is of high scientific value and application value.

Research on the microbial degradation of glyphosate is still in its early stages, and there have been no documented instances of microbial-induced heavy metal precipitation resulting from glyphosate degradation. This paper focuses on a strain of *Stenotrophomonas pavanii* MY01, which was isolated from a cornfield. The genome of this strain was mined for its glyphosate degradation mechanism. The ability of this strain to remove Cu^2+^ and Zn^2+^ was examined, and the bioremediation mechanism based on the mineral precipitation process was evaluated, and, finally, the removal mechanism of Cu^2+^ and Zn^2+^ was explored. This study introduces a novel approach for the microbial degradation of phosphorus-containing hazardous substances in agricultural fields, leading to the formation of phosphate precipitates that can capture heavy metals. It also offers a theoretical foundation and practical applications for using microorganisms to remediate soil contaminated with herbicides and heavy metals.

## 2 Materials and methods

### 2.1 Chemicals

Glyphosate (>95%) was obtained from Shanghai yuanye Bio-Technology Co., Ltd. (Shanghai, China). Methanol for high-performance liquid chromatography (≥99.9%) were purchased from YUHE NEW MAT.^1^

### 2.2 Functional strain isolation

The experiment employs the *S. pavanii*, which were initially screened by the Agricultural Pollution Prevention and Control Research Laboratory, College of Resources and Environment, Jilin Agricultural University. *Stenotrophomonas pavanii* MY01 was isolated from cornfield soil, initially the strain was an glyphosate-degrading bacterium (glyphosate: 30 mg, MgSO_4_·7H_2_O: 0.1 g, K_2_HPO_4_: 1 g, KH_2_PO_4_: lg, FeSO_4_·7H_2_O: 0.025 g, CaCl_2_: 0.025 g, H_2_O 1,000 mL, pH 7.0) (Cao et al., 2021). The isolation method used was direct isolation, i.e., the soil was taken and shaken with water. Subsequently, the mixture was directly coated on an glyphosate-inorganic salt plate, and single colonies were selected for expansion in culture. Finally, the strain *S. pavanii* MY01 was obtained. The capacity of the strain to degrade glyphosate was ascertained by means of a comparison between the protein sequence of the strain and the Query Cover of glyphosate oxidoreductase.

### 2.3 Analysis of the strain MY01 genome

For genome assembly, The filtered subreads were assembled by Canu v1.5 software, and then circlator v1.5.5 was taken to cyclizing assembly genome. For genome component prediction, Coding genes prediction was performed by Prodigal v2.6.3 (Hyatt et al., 2010). The GenBlastA v1.0.4 program was used to scan the whole genomes after masking predicted functional genes. Putative candidates were then analyzed by searching for non-mature mutations and frame-shift mutations using GeneWise v2.2.0. Transfer RNA (tRNA) genes were predicted with tRNAscan-SE v2.0 (Chan and Lowe, 2019), Ribosome RNA (rRNA) genes were predicted with Infernal v1.1.3 (Nawrocki and Eddy, 2013). Repetitive sequences were predicted using RepeatMasker v4.0.5 (Tarailo-Graovac and Chen, 2009). PhiSpy v2.3 (Akhter et al., 2012) is used for prophage prediction and CRT v1.2 (Bland et al., 2007) was used for CRISPR identification. IslandPath-DIMOB v0.2 (Bertelli and Brinkman, 2018) was used to predict genomic island in genome. antiSMASH v5.0.0 (Blin et al., 2019) was used to predict secondary metabolic gene clusters, and PromPredict v1 (Rangannan and Bansal, 2009) was used for promoter prediction.

### 2.4 Experiments on the degradation of glyphosate by strain MY01 under different conditions

The effect of different treatment conditions (including initial glyphosate concentration, temperature, initial pH, and inoculation dose) on the degradation of glyphosate by MY01was investigated in this paper, and the experimental design was as follows.

Strain MY01 was inoculated into 100 mL of LB liquid medium and incubated at 150 rpm at 30°C for 14 h (logarithmic phase). Then, the culture was centrifuged at 8,000 rpm for 5 min to collect the bacterial cells, which were subsequently washed three times with a 0.9 mol/L NaCl solution to prepare a bacterial suspension with an “OD600” of 1 for later use.

Five initial glyphosate concentrations (10 mg/L, 20 mg/L, 30 mg/L, 40 mg/L, 50 mg/L) were tested with an initial pH 7 at 30°C. Five temperatures (20°C, 25°C, 30°C, 35°C, 40°C) were tested with an initial pH of 7. Five initial pH (5, 6, 7, 8, 9) were tested at 30°C. Five inoculation doses (0.25, 0.5, 1, 1.5, 2%, w/v) were tested with an initial pH 7 at 30°C.

All experiments were performed under sterile conditions. Initial glyphosate concentrations were 50 mg/L in all experiments except for the initial concentration removal experiments. Unless otherwise specified, the inoculation dose consisted of free bacteria at a concentration of 0.5% (w/v). All experiments were performed in triplicate, and glyphosate concentrations were determined by HPLC at 1d intervals. CK is the treatment without the addition of strain MY01.

### 2.5 ANN model for optimization

The glyphosate degradation experimental results were then used to build ANN models, respectively. The input parameters of temperature, inoculation, pH, and substrate concentrations were used to construct the ANN topology. The model is done based on Matlab software.

### 2.6 Molecular docking

In this study, functional proteins that are essential for the degradation of glyphosate were subjected to molecular docking with glyphosate small molecules. Known protein sequences were obtained using the SWISS-MODEL platform and then used to obtain 3D structures.^2^ Small-molecule ligands for glyphosate in pdbqt format were obtained using pubchem software. The 3D protein structure was finally docked with the small-molecule ligands by using Autodock 4.2.6 software (Xue et al., 2022). These results were then visualized using PyMol (Zhang et al., 2024). BLAST analysis^3^ was performed to compare the sequence with the available protein sequences of the NCBI.

### 2.7 Immobilization of heavy metals Cu^2+^ and Zn^2+^

The optimal environmental conditions for the strain to degrade glyphosate were obtained from the glyphosate degradation test in the previous stage. Under these conditions, Cu^2+^ and Zn^2+^ removal tests were carried out with concentrations of the two ions set at 20 mg/L, 40 mg/L, 60 mg/L, 80 mg/L, and 100 mg/L, respectively. This was done to determine the concentration at which the strain could maximize the removal of heavy metals. In addition, the effect of glyphosate on heavy metal removal by strain MY01 was determined by adding glyphosate in concentrations of 0 mg/L, 10 mg/L, 20 mg/L, 30 mg/L, 40 mg/L, and 50 mg/L, with 0 mg/L as the control (CK).

### 2.8 Analysis of glyphosate degradation pathway and heavy metal removal mechanism of strain MY01

All the protein sequences of strain MY01 were compared with those required for glyphosate degradation on NCBI platform, and the genes with higher Query Cover and Per. Ident were retained and analyzed the glyphosate degradation pathway of strain MY01. The same approach was used to explore the removal mechanism of heavy metals Cu^2+^ and Zn^2+^.

### 2.9 Detection methods and analytical statistics

All experiments were performed in triplicate, and glyphosate concentrations were determined using high-performance liquid chromatography at 1 d intervals. The adsorption of glyphosate by strains at different stages was studied, and the upper layer of the supernatant was discarded after centrifugation. Methanol of the same quality as the supernatant was added to extract the glyphosate content of the strains, centrifugation was performed after repeated shaking, and the methanol content of the glyphosate was detected. The peak area of the removed samples minus the peak area of the adsorbed samples was used to calculate the amount of degradation. The incubation process for the heavy metal experiment was identical to that of the glyphosate degradation experiment. Following incubation, the supernatant was collected for subsequent heavy metal content detection. The concentration of glyphosate in the solution was measured by high-performance liquid chromatography (Agilent 1,260, Agilent, USA). The atomic absorption spectrophotometer was employed to quantify the Cu^2+^ and Zn^2+^ contents. The degradation rate of glyphosate and removal rate of heavy metal (Cu^2+^ and Zn^2+^) were calculated as follows (Equation 1):


(1) $$\mathrm{Removal rate}=\left(C0-Ce\right)/C0\times 100\%$$

C0 and Ce are the initial and equilibrium mass concentrations, mg/L.

The data were analyzed and plotted using Microsoft Excel 2021, Origin 2018, and Photoshop 2021. Standard errors are represented by the error lines of the three replicate tests. The neural network was fitted using MATLAB R2022a software.

## 3 Results and discussion

### 3.1 Degradation of glyphosate by strain MY01

**Figure 1:**
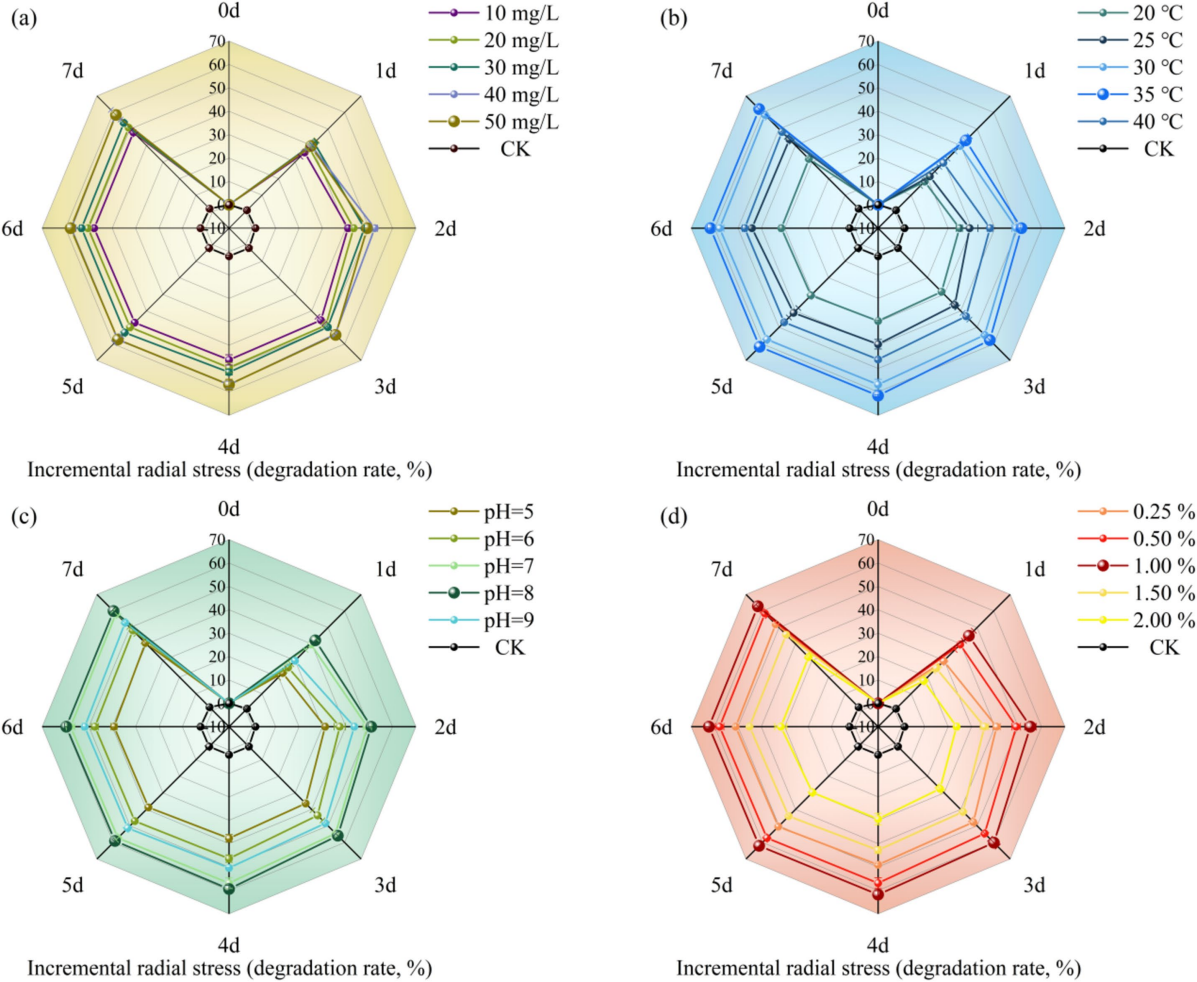
Effect of different conditions [(a) substrate concentration, (b) temperature, (c) pH, (d) inoculation] on herbicide glyphosate degradation rate of strain MY01.

The degradation effect of MY01 on glyphosate under different substrate concentration conditions is shown in Figure 1a. The experimental results revealed degradation rates of 36.42% at a substrate concentration of 1 mg/L and 48.37% at 5 mg/L over 3 days. This indicates that glyphosate degradation by the strain decreased as the substrate concentration increased. After 3 days, the degradation rate was 54.52% at a glyphosate concentration of 3 mg/L, suggesting that the strain can tolerate glyphosate and that the degradation rate remains relatively stable at concentrations up to 3 mg/L. The results also indicate that while glyphosate is a toxic herbicide, higher concentrations initially increase the degradation rate, but over time, the rate of degradation decreases. Glyphosate itself belongs to a class of toxic herbicides, and excessive addition will adversely affect the growth and reproduction of the strain. Zhang et al. found that the degradation rate of glyphosate by strain *Chryseobacterium* sp. Y16C decreased significantly when the concentration of glyphosate increased (Zhang W. et al., 2022). This is similar to the results of the current pilot study.

Temperature is a key factor influencing microbial growth rate, cellular activity, and the degradation of organic pollutants. Therefore, selecting the optimal temperature can enhance a strain’s ability to utilize the substrate effectively for growth and reproduction (Xiao et al., 2015). Figure 1b illustrates how different temperatures affect glyphosate degradation by Strain MY01. Within the temperature range of 20–40°C, the strain’s ability to degrade glyphosate initially increased with temperature, peaking at 35°C with a degradation rate of 57.66%, and then decreased at higher temperatures. The results indicate that Strain MY01’s glyphosate degradation performance is sensitive to temperature, and significant temperature fluctuations can impair microbial activity and, consequently, the degradation rate (Zhang W. et al., 2022).

pH affects microbial fitness and degradation by influencing the activity of cell membrane proteins and extracellular hydrolases (Zhang S. et al., 2022). From Figure 1c, it can be seen that strain MY01 had the highest degradation rate at pH = 8, reaching 55.90% at 3 days, and its degradation rate only increased by 2.53% compared to that at pH = 7. This indicates that strain MY01 can exhibit a more effective degradation effect in a neutral alkaline environment. Niu et al. found that (Niu et al., 2023) oligotrophic Aeromonas maltophilia can degrade pyridine more effectively in an alkaline-biased environment, which is similar to the results of the present experimental study. The abundance of degradation gene expression is a key factor influencing microbial glyphosate degradation. An alkaline environment may enhance the expression of glyphosate degradation genes in Strain MY01, leading to more effective degradation (Jiang et al., 2021). Additionally, the growth and reproduction of microorganisms produce organic acids, which lower the pH of the solution. This pH reduction can, in turn, impact the growth and reproduction of the strain.

Figure 1d shows the effect of inoculum amount on the degradation of glyphosate by strain MY01. The results showed that 1% inoculum was more suitable for strain MY01 to exert degradation of glyphosate, achieving a 60.14% degradation rate in 3 days. Degradation efficiency was lowest at 2% inoculum, achieving only 27.49% in 3 days, which was 54.29% lower than the rate at 1% inoculum. Among various factors, the inoculum amount had the most significant impact on glyphosate degradation. An excessive inoculum amount reduced the degradation rate, likely due to microorganism agglomeration, which limits the effective surface area and binding sites. Additionally, the shortened growth cycle of the strains resulted in an insufficient substrate for growth and metabolism (Xm et al., 2015). Such phenomena were also found in experiments to study microbial degradation of tunicamycin by Zhang and Wang (2021). Using a smaller microbial inoculum reduces the relative cost of removing glyphosate residues from the environment. Therefore, this study identifies 1% inoculum as the optimal concentration for effective glyphosate degradation.

### 3.2 Optimization of conditions for degradation of glyphosate by strains of bacteria based on neural network

**Figure 2:**
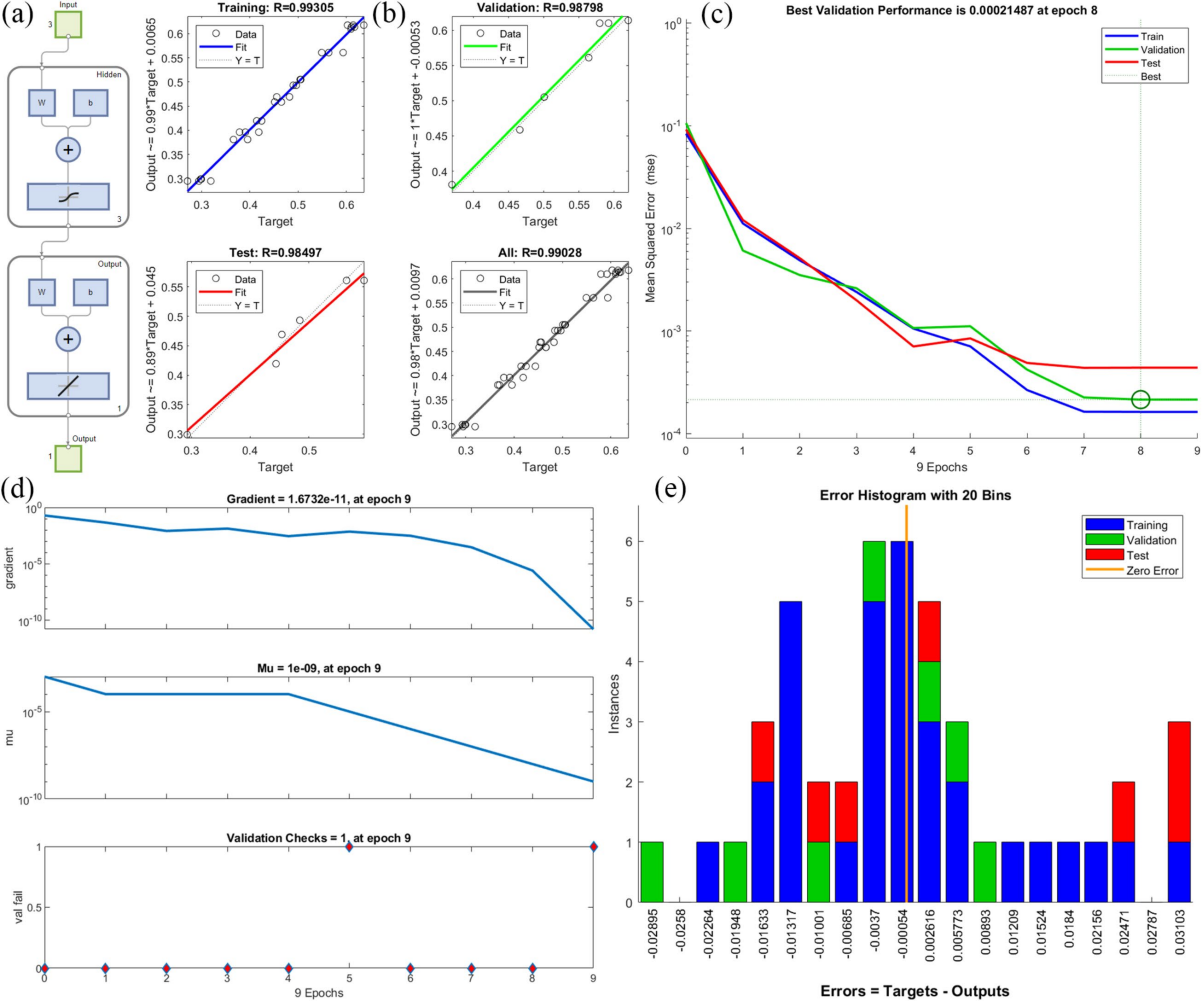
Neural network fitting to analyze the optimal conditions for the degradation of glyphosate; **(A)** The optimization of conditions for various environmental factors affecting degradation through a neural network, **(B)** Comparisons between the predicted and experimental values, **(C)** Best validation performance, **(D)** Degradation data train, **(E)** Error histograms.

ANNs have several layers of processing units. These neurons process the input data and send the transfer function to the neurons in the hidden layer (Liu et al., 2022). Figure 2 shows the optimization of conditions for various environmental factors affecting degradation through a neural network. In this experiment, the results of herbicide degradation were optimized using a neural network with three input layers, three hidden layers, and one output layer with their associated transfer functions (Figure 2a). Comparisons between the predicted and experimental values of the output variables for the training, validation, and test data, as well as the total data, are shown in Figure 2b. The R2 values were 0.99305, 0.98798, 0.98497, and 0.99208, respectively. A high R2 value indicates that the difference between experimental and predicted values of the output variable for various subsets is small. The MSE reached a minimum for the training, validation, and test data at 8 epochs (Figures 2c). Degradation data were trained for 9 epochs, respectively (Figure 2d). The gradient decreased gradually with training. The Mu parameter can determine the equilibrium relationship between the current step size and the gradient direction with each weight update. The variable ‘val fail’ indicates the number of test failures. Error histograms with 20 bins are shown in Figure 2e. Instances represent the number of data points in each error condition. The results show that the data for TEST were more concentrated on zero error compared with TRAINING and VALIDATION, indicating that the TEST error value is smaller.

### 3.3 Mechanistic investigation of glyphosate degradation by strain MY01

**Figure 3:**
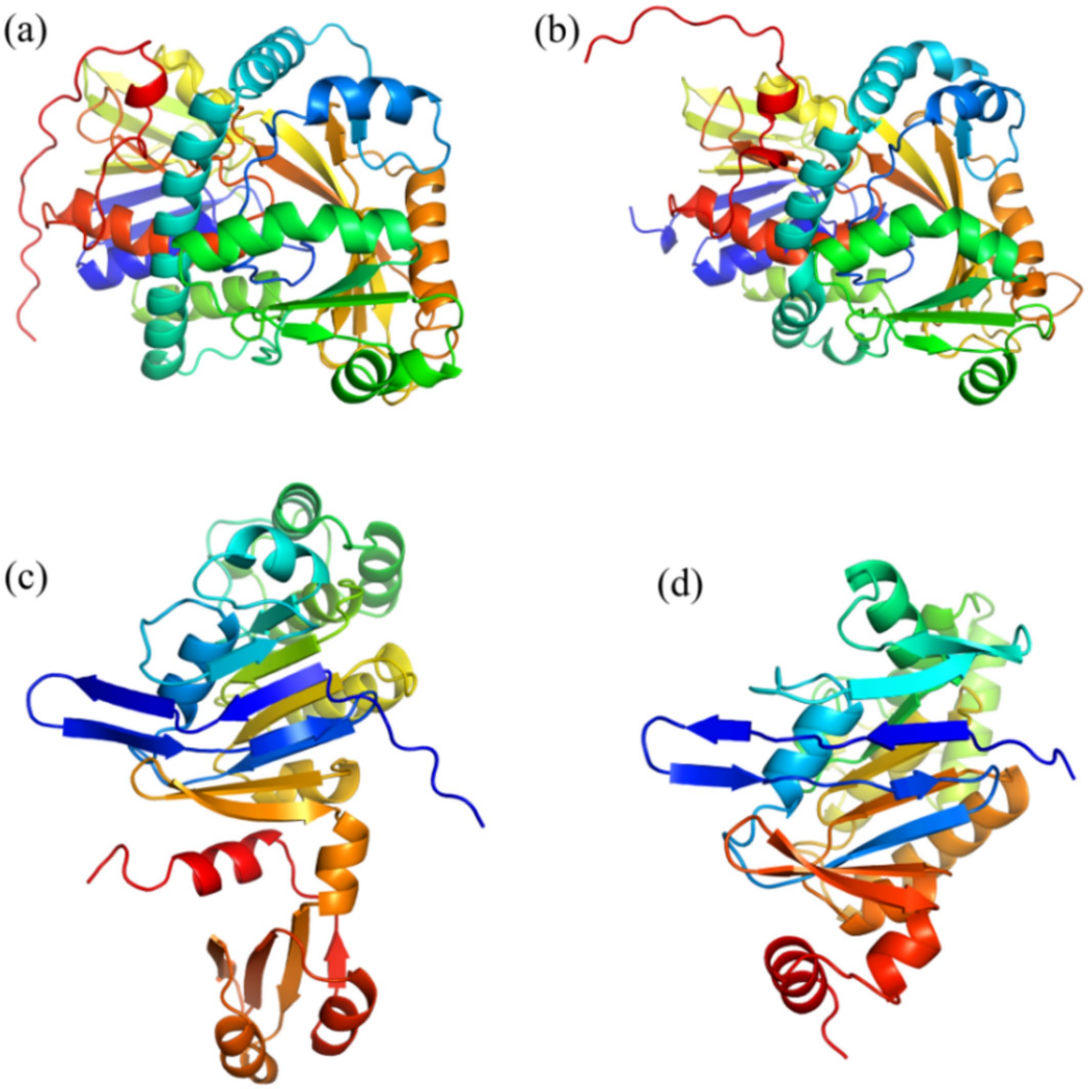
GE000474 and GE002603 with glyphosate oxidoreductase and C-P lyase comparison of sequence visualization structures; (a) GE000474, (b) glyphosate oxidoreductase, (c) GE002603, (d) C-P lyase.

**Figure 4:**
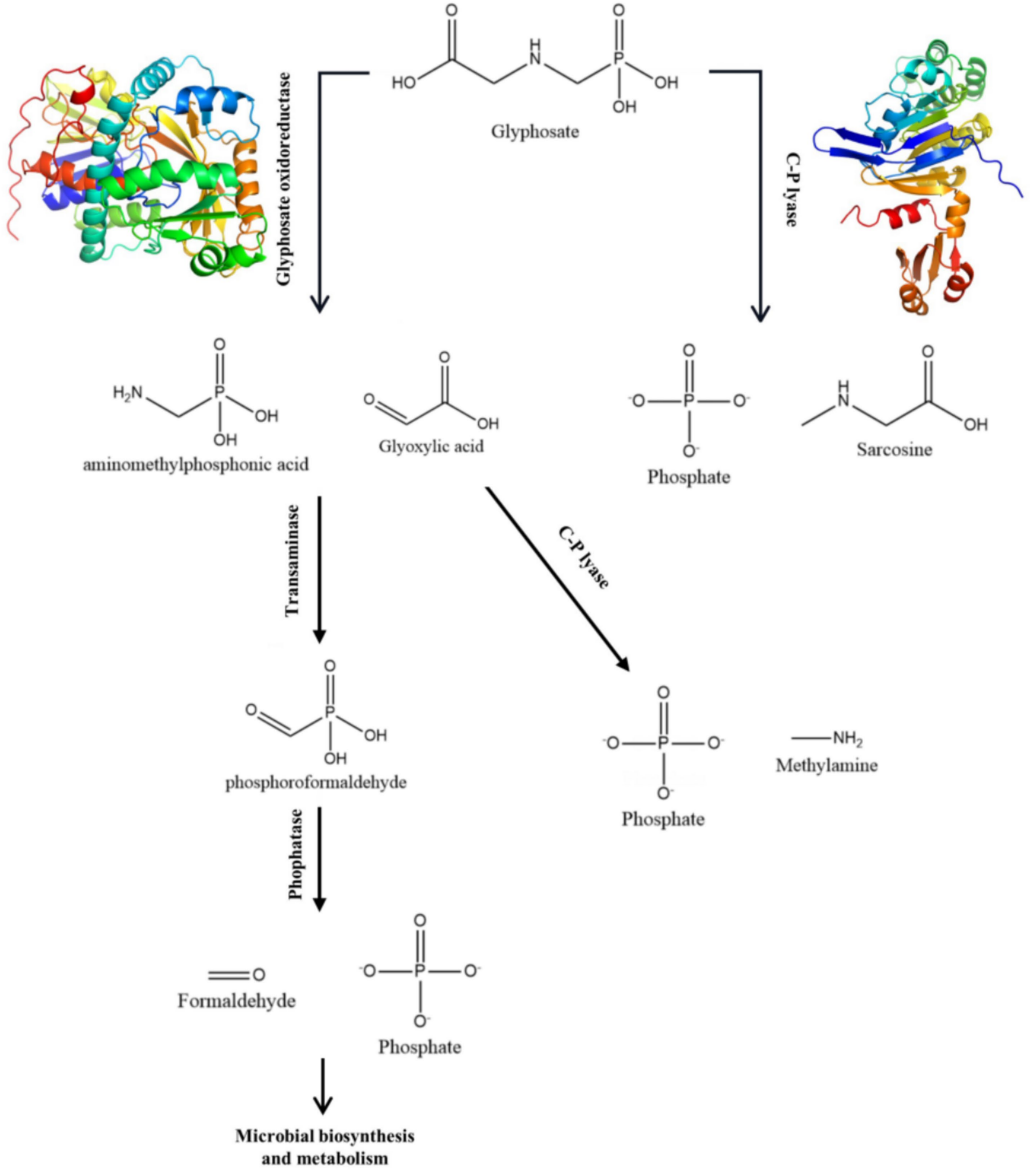
Glyphosate biodegradation pathway by strain MY01.

Table 1 shows the results of the glyphosate-degrading enzyme protein sequences compared with the total protein sequences of the MY01 genome (the protein sequence with the highest Query Cover was retained if a single enzyme protein sequence matched multiple sequences of strain MY01). The results revealed that several protein sequences from strain MY01 had over 90% Query Cover with glyphosate-degrading enzyme sequences. Glyphosate oxidoreductase, crucial for glyphosate degradation, showed that the GE000474 protein sequence of MY01 had a 96% Query Cover with the glyphosate oxidoreductase sequence. Additionally, the GE002603 protein sequence had a 97% Query Cover with the C-P lyase sequence. Figures 3a,b show the structure of the GE000474 sequence protein and the structure of glyphosate oxidoreductase. From the figure, it can be seen that the structural similarity between the two is extremely high, as well as the sequence of GE002603 and the sequence of C-P lyase (Figures 3c,d). Since protein function is determined by their structure, the high degree of similarity between the GE000474 and GE002603 sequences and the functions of glyphosate oxidoreductase and C-P lyase suggests that these sequences likely perform similar roles. This suggests that strain MY01 may be able to degrading glyphosate through two pathways, as shown in Figure 4, it can be seen that in addition to glyphosate oxidoreductase and C-P lyase, there are transaminase and phosphatase associated with the degradation of glyphosate, and these types of enzymes can degrade the degradation products of glyphosate further. Previous studies have found that glyphosate oxidoreductase and other glyphosate-degrading enzymes involved in this experiment are mostly intracellular enzymes (Mellis et al., 2021). This is the reason why strain MY01 can grow and multiply in a medium with glyphosate as the only carbon (C) and nitrogen (N) source. From the degradation pathways, it can be seen that no matter how strain MY01 degraded glyphosate, PO_4_^3−^ would be produced, which also suggests that the strain can play the function of immobilizing heavy metals while degrading glyphosate.

**Table 1:** Enzymes encoding genes in strain MY01 that may be involved in glyphosate degradation.

Protein names	Accession	GI	Gene ID	Query cover	Per. Ident.
Glyphosate oxidoreductase	ADV58259.1	319655179	GE000474	96%	25.06%
Aminomethylphosphonic acid	AAB14712.1		GE002383	95%	35.20%
C-P lyase	AEH86200.1	336026549	GE002603	97%	28.17%
Transaminase	CYV95473.1	995086780	GE003516	85%	25.29%
Phosphatase	WP_227948517.1	2128755079	GE003683	83%	30.26%

**Figure 5:**
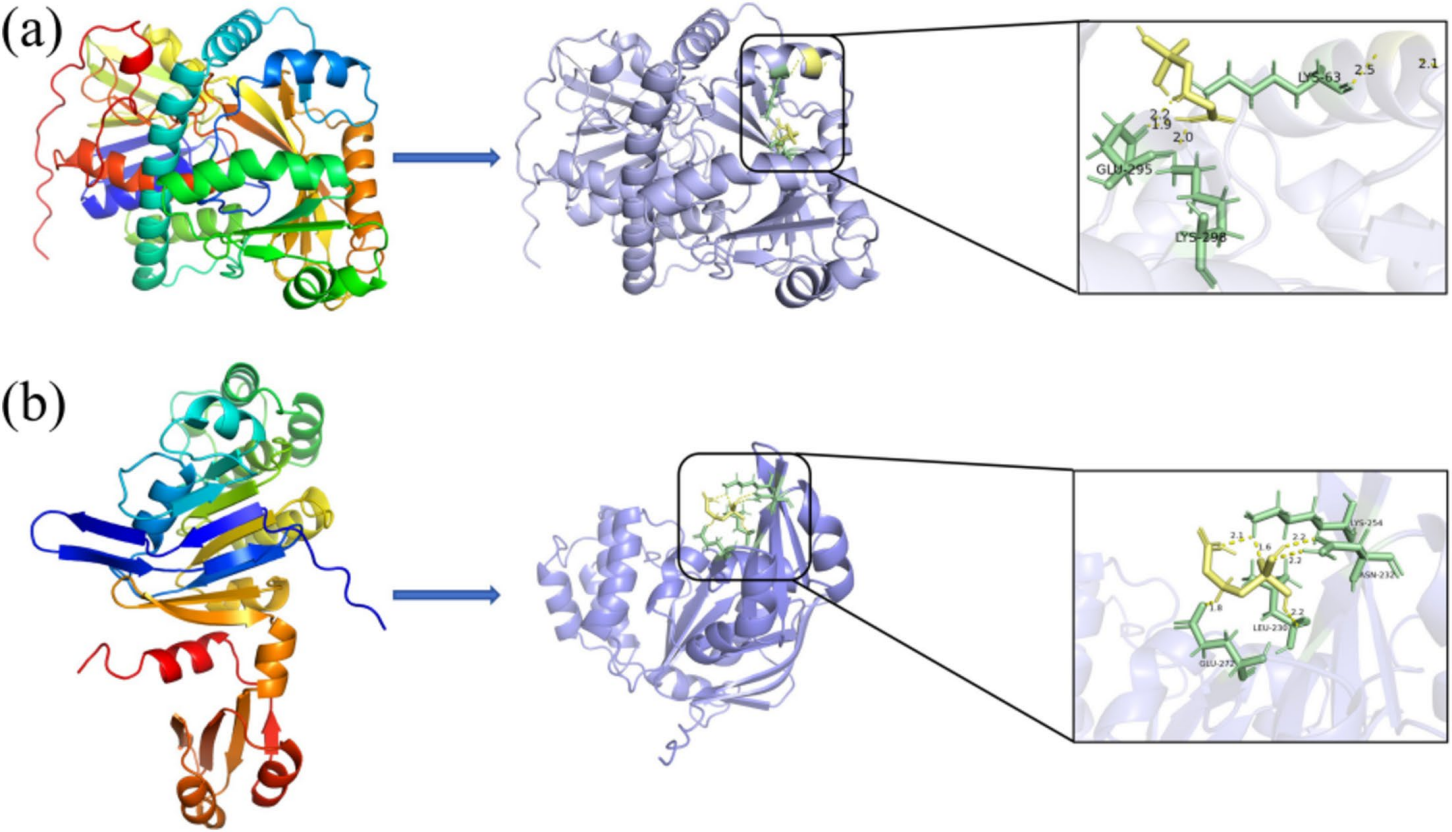
Docking diagram of GE000474 **(A)** and GE002603 **(B)** protein sequence with the molecular structure of glyphosate.

Protein docking studies unveil insights into protein-small molecule interactions and enzyme catalytic mechanisms (Zhang et al., 2023). The glyphosate structure was docked to the GE000474 protein sequence, and the results are shown in Figure 5a. It can be seen from the figure that the GE000474 protein structure can be well combined with the glyphosate molecular structure. Figure 5b shows the results of docking the GE002603 sequence to the glyphosate structure, and the two work equally well together. The binding energies between glyphosate and the two proteins were 2.94 and 3.17 kcal/mol, respectively. To better understand the factors affecting protein-ligand binding, we analyzed the interaction patterns. The results showed that glyphosate forms hydrogen bonds with GLU-295, LYS-63, and LYS-298 on the GE000474 protein, with bond strengths up to 2.5. Additionally, the GE002603 protein forms multiple hydrogen bonds with glyphosate, with bond strengths up to 2.2.

### 3.4 Removal of Cu^2+^ and Zn^2+^ by strain MY01

**Figure 6:**
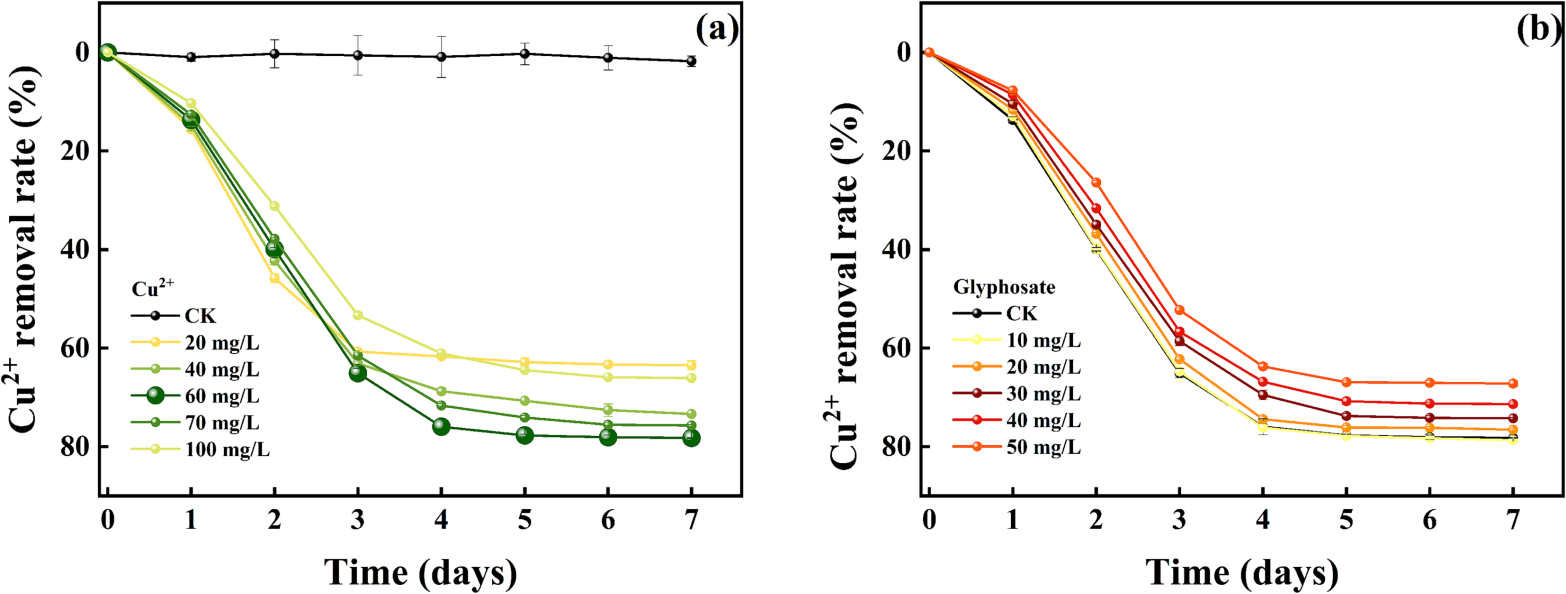
Removal of Cu^2+^ by strain MY01; (a) no glyphosate added, (b) glyphosate added.

The removal effect of strain MY01 on heavy metal Cu^2+^ before and after the addition of glyphosate is shown in Figures 6a,b. The results demonstrated that strain MY01 exhibited high resistance to Cu^2+^, maintaining normal growth and reproduction even at a concentration of 100 mg/L. Without glyphosate, strain MY01 was able to remove 61.08% of Cu^2+^ within 4 days. After 5 days, the strain had little effect on Cu^2+^ removal. In this experiment, Cu^2+^ concentrations ranged from 20 mg/L to 100 mg/L. The removal rate of Cu^2+^ initially increased with concentration up to 60 mg/L but then decreased. This is because higher Cu^2+^ concentrations make more Cu^2+^ available per unit volume, allowing the strain to remove more Cu^2+^. However, excessive Cu^2+^ concentrations inhibit the strain’s growth and reproduction, leading to a reduced removal rate once the concentration exceeds 60 mg/L. Zhao et al. found that strain *Rahnella* sp. LRP3 was unable to grow and reproduce normally at a Cu^2+^ concentration of 130 mg/L (Zhao et al., 2019). This is similar to the results of the current pilot study. Similar results were reported by Hansda et al. (2017).

The removal effects of strain MY01 on heavy metal Zn^2+^ (8a before glyphosate addition, 8b after glyphosate addition) were similar to those of Cu^2+^. The removal rates were similar between 60 and 80 mg/L of Zn^2+^, reaching 68.39 and 68.54%, respectively, at 3 days. The removal rates were significantly higher compared to other treatments. Unlike Cu^2+^, high concentrations of Zn^2+^ do not inhibit the growth and reproduction of strain MY01. The strain continued to remove Zn^2+^ effectively at concentrations up to 400 mg/L, whereas it ceased to grow at a Cu^2+^ concentration of 300 mg/L.

**Figure 7:**
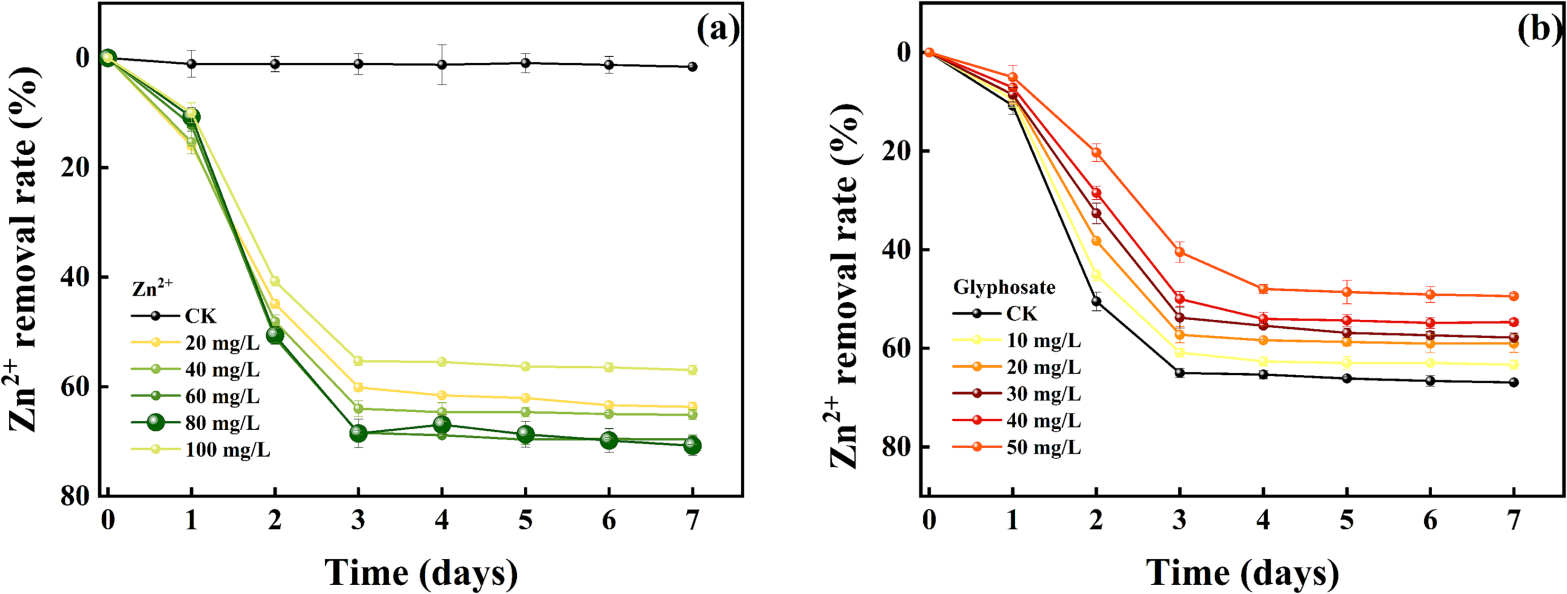
Removal of Zn^2+^ by strain MY01; (a) no glyphosate added, (b) glyphosate added.

Comparing heavy metal removal by strain MY01 with and without glyphosate showed that glyphosate addition decreased the removal efficiency in all cases. Specifically, with 50 mg/L glyphosate, the removal of 60 mg/L Cu^2+^ dropped by 14.11%, and the removal of 80 mg/L Zn^2+^ decreased by 30.18% (Figures 7a,b). The reason for this is mainly because glyphosate itself contains some toxicity and may have an inhibitory effect on the growth of the strain. Helander et al. found that glyphosate application negatively affects a wide range of soil microorganisms, such as arbuscular mycorrhizal fungi, which is similar to the findings of the present experiment (Helander et al., 2018). While the addition of glyphosate slightly reduced the microorganisms’ effectiveness in removing Cu^2+^ and Zn^2+^, it still resulted in the removal of both glyphosate and heavy metals. This combined removal is more valuable than using microorganisms alone. Additionally, after degrading glyphosate, the strain produces PO_4_^3−^, which can form stable phosphate precipitates with heavy metals, further reducing their environmental impact.

### 3.5 Mechanism of Cu^2+^ and Zn^2+^ removal by strain MY01

In this experiment, all genes in strain MY01 related to resistance and removal of Cu^2+^ and Zn^2+^ were analyzed, with the results presented in Supplementary Tables S1, S2. Copper-exporting P-type ATPases are proteins that facilitate the efflux of intracellular Cu^2+^. These proteins are crucial for maintaining copper ion balance within the cell and preventing the toxic accumulation of copper ions by expelling them from the cell (Radford et al., 2003). Copper-transporting ATPase is functionally similar and is mainly responsible for intracellular Cu^2+^ transport and distribution to ensure intracellular copper homeostasis and rational distribution. Copper-exporting P-type ATPase B, also known as ATP7B, is a protein belonging to the P-type ATPase family and is also involved in the copper ion transport and distribution (Singleton et al., 2010). Cation efflux system protein CusA is a membrane protein found in bacteria that plays a key role in resistance to heavy metal cations, especially copper and silver (Puthusseri et al., 2021). The cation efflux system protein CusC is closely related to metal ion tolerance and efflux, and its main function is to cooperate with other members (Cation efflux system protein CusA, Cation efflux system protein CusB, Cation efflux system protein CusF, etc.) in assisting in the detection, binding, and transport of heavy metal ions, especially copper and silver, for efflux from bacterial cells (Bao et al., 2017). Copper resistance protein A and copper resistance protein B are usually part of the bacteria and help the bacteria to resist high concentrations of copper ions (Hennaux et al., 2022). Copper chaperone helps reduce the toxicity of excess copper ions in cells by forming stable complexes with copper ions and assisting in the transport of copper from one place to another in the cell (Grasso et al., 2021). High-affinity zinc uptake system ATP-binding protein ZnuC and Zinc import ATP-binding protein ZnuC play an important role in intracellular zinc ion uptake and transport, which helps to maintain intracellular zinc ion homeostasis to prevent zinc concentration from ZnuC is important in the uptake and transport of zinc ions in the cell and helps to maintain the balance of zinc ions in the cell to prevent the cell from being adversely affected by high or low zinc concentrations (Ilari et al., 2014). Zinc-exporting P-type ATPase is mainly responsible for removing Zn^2+^ from the cell to help maintain intracellular zinc ion homeostasis (Bækgaard et al., 2010). The above analysis shows that strain MY01 has a variety of proteins that are resistant to Cu^2+^ and Zn^2+^ and can remove them. Because of this, strain MY01 can be grown and propagated in a high concentration of Cu^2+^ and Zn^2+^ environment and can effectively remove the excess Cu^2+^ and Zn^2+^ from the environment.

**Figure 8:**
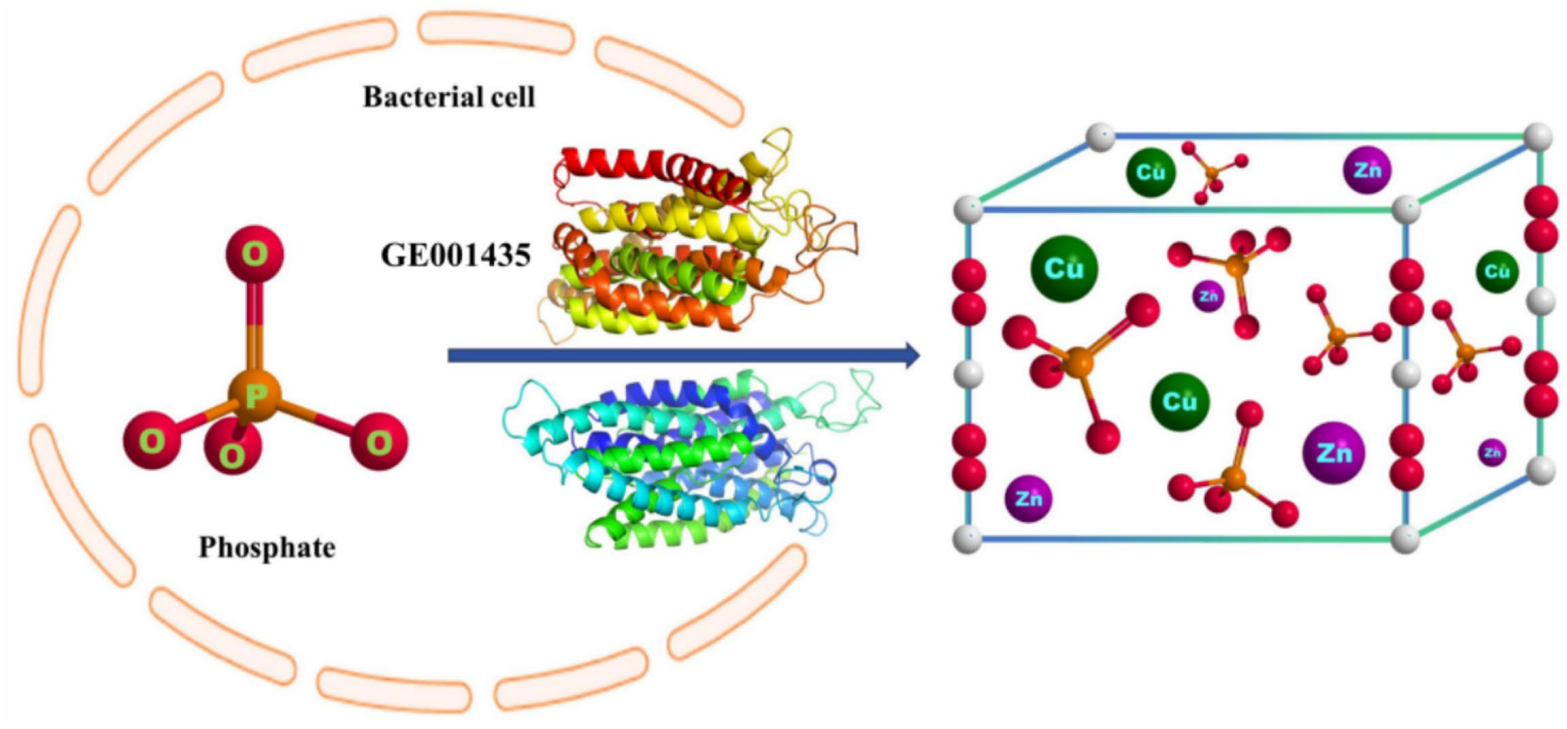
The mechanism of heavy metal-producing phosphate precipitation induced by strain MY01 is described herein.

Microbial-induced precipitation of heavy metals Cu^2+^ and Zn^2+^ is mainly due to the microbial generation of CO_3_^2−^ or PO_4_^3−^ to form stable coordination compounds with heavy metals (Zeng et al., 2017). The microbial degradation product of glyphosate in this experiment was PO_4_^3−^ (Figure 4), so the main component of the precipitate was phosphate. After NCBI platform comparison, the protein sequence GE001435 was found to have a very high similarity to the phosphate pathway, and the results of this sequence visualization and the mechanism of heavy metal Cu^2+^ and Zn^2+^ precipitation induced by strain MY01 are shown in Figure 8.

### 3.6 Genomic analysis of strain MY01

The genome sequencing data of strain MY01 was assembled to obtain the 0 Gap genome completion map. The complete genome of the strain is 4,542,518 bp with 66.84% GC content, There are 600 genes related to metabolism, Amino acid transport and metabolism genes 208, Nucleotide transport and metabolism genes 61, Carbohydrate transport and metabolism genes 138, Coenzyme transport and metabolism genes 86, Lipid transport and metabolism genes 107.

## 4 Conclusion

1. The optimal conditions for glyphosate degradation by strain MY01 were determined to be 34°C, pH 7.7, and an inoculum concentration of 0.7%, achieving a maximum glyphosate degradation of 72.98% within 3 days, as predicted by neural network modeling. The sequence analysis revealed that GE000474 and GE002603 had high similarity to glyphosate oxidoreductase and C-P lyase, with similarities of 96 and 97%, respectively. Strain MY01 degrades glyphosate via three pathways: (1) glyphosate oxidoreductase, transaminase, and phosphatase converting glyphosate to formaldehyde and PO_4_^3−^; (2) glyphosate oxidoreductase and C-P lyase converting glyphosate to methylamine and PO_4_^3−^; and (3) C-P lyase converting glyphosate to sarcosine and PO_4_^3−^. Molecular docking of the GE000474 and GE002603 protein sequences with glyphosate demonstrated effective binding, with binding energies of 2.94 kcal/mol for GE000474 and 3.17 kcal/mol for GE002603.
2. The results demonstrated that strain MY01 effectively removed Cu^2+^ and Zn^2+^, achieving a 75.95% removal rate at a Cu^2+^ concentration of 60 mg/L over 4 days, and a 68.54% removal rate at a Zn^2+^ concentration of 80 mg/L over 3 days. Comparison of MY01’s protein sequences with those involved in the phosphate pathway revealed that GE001435 closely resembles phosphate pathway proteins. This suggests that GE001435 likely functions similarly to these proteins, potentially facilitating the discharge of phosphates from the pathway, which then bind with heavy metals to form precipitates.

## Data Availability

The datasets generated and/or analyzed during the current study are available in the [National Center for Biotechnology Information] repository, [TaxID: 3236839]. Strain MY01 GenBank: OR258343. Strain MY01 genome accession: PRJNA1137265 (data source: www.biomarker.com.cn).

## Footnotes

^1^ http://www.yuhexincai.com

^2^ https://swissmodel.expasy.org

^3^ https://blast.ncbi.nlm.nih.gov
