# Supplementary material for: Stenotrophomonas pavanii MY01 induces phosphate precipitation of Cu(II) and Zn(II) by degrading glyphosate: performance, pathway and possible genes involved
Source: Front Microbiol. 2024 Oct 23;15:1479902. doi: 10.3389/fmicb.2024.1479902 (PMC11538021; doi:10.3389/fmicb.2024.1479902)
Supplement: Supplementary file 1 [file Data_Sheet_1.docx]

>GE000474

MRVLVLGSGVIGTTSAWYLRQAGFEVTVIDRQPGPALETSFANAGQLSFGYTSPWAAPGVPKKAIGWLFEKHAPLAIKPGMDLAQYRWLWQMLRNCTHERYAINKARMVRMSEYSRDCLNELRAQIGIEFEGRDLGTTQLFRTQQQLDASAQDIEILAQYGVPYEVLDRAGIIQAEPALAHVDGLVGALRLPRDQTGDCQLFTRRLAQMCVDAGVEFRFDQDITGLEFDGDRITGVRIDGKRETADRFVVALGSYSPALVAPLGMRLPVYPLKGYSLTLPITDPAMAPTSTILDESYKVAVTRFDDRIRVGGMAEVAGFDLSLSQRRRETLELVVSDLYPKGGDLSRAQFWTGLRPATPDGTPVIGATPFRNLYLNTGHGTLGWTMACGSGRYLADLMSARQPQISTEGLDIFRYGQYGHAPQHENRTCVLPAR

>GE002383

MIISASTDYRAAAQRRLPPFLFHYIDGGAYAEHTLKRNVSDLSDIALRQRILRNMSDLSLETELFGETLAMPVALAPVGLTGMYARRGEVQAARAADSRGIPFTLSTVSVCPIEEVAPAIQRPMWFQLYVLRDRGFMRNALERAQAAGVTTLVFTVDMPVPGARYRDAHSGMSGPNASLRRIGQAVTHPHWAWDVGLFGRPHDLGNISTYRGNPTGLEDYIGWLGSNFDPSISWKDLEWIREFWKGPMVIKGILDPDDARDAVRFGADGIVVSNHGGRQLDGVLSTARALPAIADAVQGDLKILADSGIRTGLDVVRMLALGADTVLLGRAFVYALAAQGEAGVANLLDLIAKEMRVAMTLTGAHRIADISRDSLVNLP

>GE002603

MNSTASEPVITARGLRKAYKTTVALDNASFSIPSGRIVGLIGPNGAGKTTALKAILGLTSVEGELSVLGRDPRQHRDALMNDICFIADVAVLPRWLKVREAIDFVAGVHPRFDRARCERFLANTKLQPKQRVRELSKGMIVQLHLALVMAIDARILVLDEPTLGLDILYRKEFYQRLLEDYFDEQKTIIVTTHQVEEIEHILSDVMFIRDGRIVLDAEMDDVGQRYTELLVNADQLETARALKPIDERSLTFGKTVMLFDGVPRTQLSTLGETRSPGLADLFVAIMKGTYA

>GE003516

MSTPPHTPGYSRRSHEIAPFHVMSLLARAQALEQAGHDVIHLEIGEPDFTTAEPVVRAGQAALAAGHTRYTAARGLPALRQAISGFYRSHYGLDVDPERILVTPGGSGALLLASSLLVDPGRHWLLADPGYPCNRHFLRLVEGGAQLVPVGPDTAYQLTPSLVEQHWNDDSVGALVASPANPTGTVLSADELAALSQALHARGGHLVVDEIYHGLTYGLDAPSVLQVDDSAFVLNSFSKYFGMTGWRLGWLVAPPAAVPDLEKLAQNLYISASSIAQHAALACFSEESMAIFEQRREAFRQRRDFLLPALRELGFRIEVEPQGAFYLYADVSAFTDDAQAFCAHFLETEHVAFTPGLDFGFHRANQHVRLAYTQQVPRLQEAVARIARGLKRWQGSR

>GE003683

MATLAASPLAGEGRPWRRALLWLALLGPFFFASYGFANWMAGRHATLPVMAFGWETRIPFVPWTIVPYWSIDLFYAVSFFLCRQRLELDRHALRLFSAQLIAVSCFLLWPLRFSFERPQIGGVFGWLFDVLLGFDKPFNQAPSLHIVLLIVLWVKFAQYLHGVWRWVLHAWALLIGVSVLTTFQHHFIDIPTGLLAGWLCVWLWPEQGTPPLRAWRTARDAKRWRLAGVYLLGAAVLLVPVVLLRGAALWLLWPVVSLLLVALAYAGLGTAVFQKRADGRLTMAARWLLAPYLGAAWVNSRLWTRRAPHPVPVMDDVWLGRIPCAALPTPLVGVVDTCAELSCRAPGAAYASVPMLDLVVPTPEQLREAADAIERLRVQGPLLVCCALGYSRSAASVATWLLRSGRAADANAAVAIVRAARPGIVLHAAHLHAITAAAGARA

>GE001808

MSTPRATAAPASPATISLPIEGMTCASCVGRVEAALSRVEGVGSVSVNLATERADIRPSGPVDRAALIQAVERVGYDVPAATTELSVEGMTCASCVGRVERALLAVPGVSQASVNLATERATVRGVAEVAALVAAIDKAGYDARVIEAGVQSDDEAAEKKDAERAELKRDLIVASALALPVFVLEMGSHLIPGMHEWVMSTIGMQASWYLQFVLTALVLAIPGRRFYQKGIPALLRLAPDMNSLVAVGTAAAFGYSVVATFLPTLLPAGTVNVYYEAAAVIVALILLGRFLEARAKGRTSEAIKRLVNLQAKVAHVVRDGRTVDVPVNEVLSGDVVEVRPGERVPVDGEVVEGRSYIDESMISGEPIPVEKQPGSSVVGGTVNQKGALTVRATAVGAQTMLAQIIRMVEQAQGSKLPIQAVVDKVTLWFVPAVMLAALATFAVWLIFGPSPALSFALVNAVAVLIIACPCAMGLATPTSIMVGTGRGAEMGVLFRKGEALQLLKDAQVVAVDKTGTLTEGRPRLTDLEIAAGFDHNTVLAAVAATESRSEHPIARAIVDAATGEGIALPGMVDFESVTGMGVRASVEGARVEVGADRFMRDLGVDITVFATLAAELGIQGKSPLYAAIDGRLAAIIAVSDPIKPSTPAAIAALHQLGLKVAMITGDNAGTAQAIARQLGIDDVVAEVLPEGKVEAVRRLKATHGHVAFVGDGINDAPALAEADVGLAIGTGTDIAVESADVVLMSGNLQGVPNAIALSKATLGNIRQNLFWAFAYNTALIPVAAGVLYPTWGVLLSPVFAAGAMALSSVFVLGNALRLRRFQPPMADAPAAIH

>GE002202

MIDRLIAYCLRQPLMVMLALLLFIGAGIAAFRVLPVEAFPDVSDTQVTVVSLHPGRAAEEVEREVTMPLEVALSGIPHSVRVFSHTQFGLSMIILTFDDKADDYFARQQVLERLQGVELPEGVTPELEAMSSAVGEIYRYVLKGPHLTPTQLRTVQEWTMERSLRTVPGVADVISFGGFARTFQVKPDLDKLRDRGISLAEFAEALEKGSSNAGGGYVERGQQQFLIRGVGLMRSPADIGNVVVAQRSGTPILVRDLAGIADTGLPRQGLVGQDDNDDAVFGMVLMRKGENPSDVLDALHARIAEIEANQLPAGVSIEPFYDRSWLVSTTLKTVFSNLLEGAVLVFLVLWLFLYNARAALIVAAMMPLALLSTFLGLHLWGVPANLLSLGAMDFGIIIDGAVIVTEHIVSRLSKLPPAADRKTRFSTILSAASEVGRPTFFSMLIIIAAHIPIFTLQRQEGRMFAPMAYSVTSALIGALILALTVVPLFCYRWLRRDRMRDGNPLMDRLTGWYRPVLERALARPRAVVLTAVALLVGTLALGTRLGSEFLPELDEGSIWLTATLDPSTSLGEAQQQSRRIRELVGTFPQVSTVVAKLGRPEDGSDAKGANQIEALVALKPEKEWPKGVGKRQLVSDLQRTLEQRIPGPEFSISQPVRDNILESISQIKGQVVIKVSGSDLDVLNQQAQAILGQVRGVEGVESAFIDRDGSLPQLQIEIDRDRAARYGLNVRDVDEVIETALGGRQVGELWEGDRRFPITLRLDDADRDLQRLRTVPVGIGDGHTVTLSDVADFRMASGAINISRENAQRVKAVSIFIAGRDMGSVVADMRKRVDASVQLPEGYRLEWSGEFENQQRAMKRLGWVIPLSVLIIFVLLFDAFKDISSAALILANVPLAMIGGILALWLTGIPLSVSAAIGFIALFGQAVLNGVVMLSRFAQLREQGMDLLQSVVQGSLQRLRTVLMTALLAMFGLLPMATSHAIGAETQKPLAVVVIGGLLSAMLLTLLVLPTLYYWVHRRREARIA

>GE003871

MKPMLLRALAAATMTTVLGGCVSMAPHYQRPEAPVPAQFANAATGETGPALAMPAWREVFLEPRLQQVIALALQNNRDLRVAVLQVEKERAQYRIQRAALLPSVDASGSVTRSRVSDANSEIGVSQVTESDAVQVGFSSWELDLFGRIRSLKNEALQNWLASAENQRAARTSLVAEVAKAWLALAADEQSLAFTRQTLDSQQQTLQRTEARHAQGLASGLDLSQVQTSVDAARAALARLQTQQAQDRDALQLLVGAPLDAALLPSAQALDGTVALAPLPSNLPSSVLLQRPDVLSAEHALQAANADIGAARAAFFPTLTLTANYGHSSTALSTLFSAGTRGWSFAPSITAPIFHAGALKASLDASKIGKDIGIAQYEKAIQQAFSEVADALATRDDLGAQMEAQRALVAASQRSYTLAEARYRTGLDGYLQALDAQRSLFAAQQDLIALQQQEAGNRVTLFKVLGGGADAR

>GE003088

MNTRIPPGPGAVPMPSRRLFVQGLAAGGVVAGIAAVGVPQRALAAATATPRLAGAPAVLSDTRIELAIGESLANFTGRTRPAITVNGSLPAPILRWREGQTVDLFVRNTLERHPTSIHWHGILLPANMDGVPGLSFNGIGPGETYHYHFQLKQSGTYWYHSHSMFQEQAGLYGALIIDPAEPAPYRHDREHVIMLSDWTDMDPGALFRRMKKLAEHDNYYKRTLPDFLRDVKRGGWSAALSDRGMWGRMRMTPTDISDINAHTYTYLMNGTAPAGNWTGLFRSGEKVLLRFINGASMTYFDVRIPGLKMTVVAADGQYIHPVSIDEFRIAPAETYDVLVEPTGQDAFTIFCQDMGRTGFAAGTLAVRHGLQAPIPERDPRPLLTMSDMGHDMGSGGHGGHDMAAMKGMEGGCGASMGHGAPAGGDTASKAPKHPASERNNPLVDMQSSATEPKLDDPGIGLRDNGRQVLTYGAMRSLFEDPDGREPSREIELHLTGHMEKFSWSFDGIPFASAEPLRLNYGERMRIVLVNDTMMQHPIHLHGVWSDLENAQEEFQVRKHTIDMPPGTRRSYRVRADALGRWAYHCHLLYHMEAGMMREVRIEE

>GE003089

MSRSLLSPSLLALGLAAALPVFAQSHAGHDMAAMDPPAKTANAPVEQADHSKMDHSKMDSGATDHAGMDHSKMDHGAMQPAAMDHSQMDHGAMGHATPAPTEPRDPIPVPSDADRAAAFPPIAHGAMEHAPEINSLLLIDRLEHWDGKSSNGQAWEATGWIGGNINRLWLRTDGERSRGRTESSSLEALYGRSVSPWWDVLVGVRQDFRPADSRTWAAIGIQGLAPYKFESSATLYMGSGGQVLAKAEVEYDVLLTNRLILQPLLEATVAARDEPEYGIGRGLNKVEAGLRLRYEFSRRFAPYIGISHERTFSDTADYAGDHARDTRWVAGVRMWF

>GE001810

MEFHVDGMTCGGCARSVTRAIQQIDPNASVVADPPTGLVKVQTTASQEQVFAAVNDAGFPPRTA

>GE001612

MPLITLQNVDFSVGGPLLLEKAELSIEPGERIALIGRNGAGKSTLLKLLSGDHKPDDGEVRVQQGVRVTRLEQEVPHGAAGSVFDVVADGLGELGQWLAEFHHLSHAEVFDGEALGNVQAKIDAANGWGLDQRVSETLTKLDLDGDAEFGRLSGGMKRRVLLARALVSSPDVLLLDEPTNHLDIEAIDWLEAFLKGWSGSVVFVTHDRRFLRALATRIVEIDRGQVTSWPGDWANYERRREERLNAQAQENARFDKLLAQEEVWIRQGIKARRTRDEGRVRRLKAMRTERSQRRDLSGNVKMEAAQGVSSGKKVIDVKDISFAFGERTMVRDFTTTILRGDRIGLIGPNGSGKTTLLKLLLGELQPAKGEVNAGTNLQIAYFDQYRAVLREDWSAIENVAEGRDFLEFNGKRKHVHAYLQDFMFTPERARAPITRLSGGERNRLLLAKLFAQPSNLLVMDEPTNDLDVETLELLEELLGEYTGTLLLVSHDRDFIDNVVTSTLVMEGDGVIGEYVGGYSDWQRYAASVAAPAAAPAAKPATAAPAAAAAATPAAPKRKLAYKEARELEQLPKTIEKLEGDVEGLTSAMNDPSFYTRSSAEVTAHTQQLAKVQAELDAAYARWEELEG

>GE001974

MSVLLKLHEVVVRRQQREILHGISLAFEPGTVTALVGPNGAGKSTLLAVAAGDLRADAGEVSLLGKPLASYKAGPLARERAVMPQEHGVRFAFSVEEVVAMGRLPHSPDPRVDDARVEAAIDAAELQALRLREVQQLSGGESARTTFARVLAQDTPLLLLDEPTAALDLRHQERTLRSVRACAEAGACVIVVLHDLNLAAGYADRIVLLEQGRVAADGTPLQVLTEDNLQRVYQQDVVVLEHPRRGVPLVVVT

>GE001808

MSTPRATAAPASPATISLPIEGMTCASCVGRVEAALSRVEGVGSVSVNLATERADIRPSGPVDRAALIQAVERVGYDVPAATTELSVEGMTCASCVGRVERALLAVPGVSQASVNLATERATVRGVAEVAALVAAIDKAGYDARVIEAGVQSDDEAAEKKDAERAELKRDLIVASALALPVFVLEMGSHLIPGMHEWVMSTIGMQASWYLQFVLTALVLAIPGRRFYQKGIPALLRLAPDMNSLVAVGTAAAFGYSVVATFLPTLLPAGTVNVYYEAAAVIVALILLGRFLEARAKGRTSEAIKRLVNLQAKVAHVVRDGRTVDVPVNEVLSGDVVEVRPGERVPVDGEVVEGRSYIDESMISGEPIPVEKQPGSSVVGGTVNQKGALTVRATAVGAQTMLAQIIRMVEQAQGSKLPIQAVVDKVTLWFVPAVMLAALATFAVWLIFGPSPALSFALVNAVAVLIIACPCAMGLATPTSIMVGTGRGAEMGVLFRKGEALQLLKDAQVVAVDKTGTLTEGRPRLTDLEIAAGFDHNTVLAAVAATESRSEHPIARAIVDAATGEGIALPGMVDFESVTGMGVRASVEGARVEVGADRFMRDLGVDITVFATLAAELGIQGKSPLYAAIDGRLAAIIAVSDPIKPSTPAAIAALHQLGLKVAMITGDNAGTAQAIARQLGIDDVVAEVLPEGKVEAVRRLKATHGHVAFVGDGINDAPALAEADVGLAIGTGTDIAVESADVVLMSGNLQGVPNAIALSKATLGNIRQNLFWAFAYNTALIPVAAGVLYPTWGVLLSPVFAAGAMALSSVFVLGNALRLRRFQPPMADAPAAIH

Table S1. Enzymes encoding genes in strain MY01 that may be involved in Cu^2+^ removal

| Protein Names | Accession | GI | Gene ID | Query Cover | Per. Ident |
| --- | --- | --- | --- | --- | --- |
| Copper-exporting P-type ATPase | CAG7586645.1 | 2063913326 | GE001808 | 94% | 41.05% |
| Copper-transporting ATPase | EMB84348.1 | 449182316 | [GE001808](https://blast.ncbi.nlm.nih.gov/Blast.cgi#alnHdr_Query_91465) | 98% | 42.28% |
| Copper-exporting P-type ATPase B | EEL13892.1 | 228658151 | [GE001808](https://blast.ncbi.nlm.nih.gov/Blast.cgi#alnHdr_Query_91465) | 99% | 37.30% |
| Cation efflux system protein CusA | CFU82352.1 | 831199532 | [GE002202](https://blast.ncbi.nlm.nih.gov/Blast.cgi?CMD=Get&VIEW_RESULTS=FromRes&RID=K69NTWZ8114&UNIQ_OBJ_NAME=A_SearchResults_1qu0cc_1l24_dorFWKQKFEc_GTMVb_1kh4nK&QUERY_INDEX=0#alnHdr_Query_123421) | 99% | 35.24% |
| Cation efflux system protein CusC | EFO55593.1 | 308118331 | GE003871 | 99% | 39.13% |
| Copper resistance protein A | CRI56483.1 | 820683986 | GE003088 | 99% | 61.25% |
| Copper resistance protein B | QGJ33877.1 | 1775842793 | GE003089 | 92% | 48.01% |
| Copper chaperone | KAF2775170.1 | 1812038447 | GE001810 | 81% | 36.21% |

Table S2. Enzymes encoding genes in strain MY01 that may be involved in Zn^2+^ removal

| Protein Names | Accession | GI | Gene ID | Query Cover | Per. Ident |
| --- | --- | --- | --- | --- | --- |
| High-affinity zinc uptake system ATP-binding protein ZnuC | KKJ79427.1 | 816202706 | [GE001612](https://blast.ncbi.nlm.nih.gov/Blast.cgi?CMD=Get&VIEW_RESULTS=FromRes&RID=K6K5NK3P11N&UNIQ_OBJ_NAME=A_SearchResults_1qu39R_2rnx_dp9SS8WWYD3_23tp8J_1zymA6&QUERY_INDEX=0#alnHdr_Query_268709) | 94% | 28.93% |
| Zinc import ATP-binding protein ZnuC | CEI14377.1 | 734479278 | GE001974 | 81% | 30.93% |
| Zinc-exporting P-type ATPase | ABZ99206.1 | 167780908 | [GE001808](https://blast.ncbi.nlm.nih.gov/Blast.cgi?CMD=Get&VIEW_RESULTS=FromRes&RID=K6RCSMCT114&UNIQ_OBJ_NAME=A_SearchResults_1qu4HB_2AZE_dqF3RepE8Ve_GTJ71_VauBk&QUERY_INDEX=0#alnHdr_Query_71323) | 92% | 27.68% |
